# Protective effects of selenium on Bisphenol A-induced oxidative stress in mouse testicular mitochondria and sperm motility

**DOI:** 10.5935/1518-0557.20210010

**Published:** 2021

**Authors:** Zeinab Rafiee, Fatemeh Rezaee-Tazangi, Leila Zeidooni, Hadis Alidadi, Layasadat Khorsandi

**Affiliations:** 1 Student Research committee, Ahvaz Jundishapur University of Medical Sciences, Ahvaz, Iran; 2 Department of Anatomical Sciences, Ahvaz Jundishapur University of Medical Sciences, Ahvaz, Iran; 3 Toxicology Research Center, Medical Basic Sciences Research Institute, Ahvaz Jundishapur University of Medical Sciences, Ahvaz, Iran; 4 Cellular and Molecular Research Center, Medical Basic Sciences Research Institute, Ahvaz Jundishapur University of Medical Sciences, Ahvaz, Iran

**Keywords:** selenium, sperm motility, oxidative stress, Bisphenol A

## Abstract

**Objective:**

This study aimed to explore the impact of selenium (SE) on Bisphenol-A (BPA)-exposed sperm and isolated testicular mitochondria of mice.

**Methods:**

Mouse sperm and isolated mitochondria were exposed to BPA (0.8 mM) and different concentrations of SE (50, 100, and 200 µM) for four hours. The viability of sperm and isolated mitochondria as well as the mitochondrial membrane potential (MMP) were evaluated. SOD (superoxide dismutase), GSH (glutathione), MDA (malondialdehyde), and ROS (reactive oxygen species) levels in testicular mitochondria were also examined.

**Results:**

BPA concentration-dependently enhanced ROS and MDA levels in isolated mitochondria, while MMP and acclivity of GSH and SOD significantly reduced. BPA also considerably impaired spermatozoa survival and motility. SE concentration-dependently reduced mitochondrial oxidative stress, MMP, sperm survival, and total sperm motility.

**Conclusions:**

Our findings collectively suggested that SE concentration-dependently reversed BPA-caused mitochondrial toxicity and reduced sperm motility by suppressing oxidative stress.

## INTRODUCTION

Bisphenol A (BPA), an industrial chemical, is used to produce polycarbonate (a clear and hard, plastic) and epoxy resins (lining on the inside of beverage cans and metal-based foods (Mikołajewska *et al*., 2015). BPA is also used in the manufacturing of toys, eyeglasses, lenses, compact discs, thermal paper, and dental sealants (Löfroth *et al*., 2019). BPA penetrates the body through ingestion, dermal contact or inhalation (Konieczna *et al*., 2015). The exposure of humans to BPA has been related to biological systems, environmental BPA, and food intake (Engel *et al*., 2014). BPA has been found in amniotic fluid, semen, plasma, urine, and breast milk (Ikezuki *et al*., 2002).

Evidence indicates that BPA has deleterious effects on the reproductive system (Anjum *et al*., 2011; Ullah *et al*., 2018). BPA diminishes the weight of the epididymis and testicles and impairs sperm quality of rodents (Chitra *et al*., 2003; Tyl *et al*., 2008; Knez *et al*., 2014). Moreover, BPA impairs mitochondrial function by diminishing ATP, reducing mitochondria mass, and disrupting the mitochondrial membrane potential (MMP) (Kaur *et al*., 2018). Mitochondrial dysfunction affects sperm production and spermatozoa motility (Kamali Sangani *et al*., 2017). Besides, BPA diminishes antioxidant levels and stimulates ROS generation in testicular tissue (Chitra *et al*., 2003).

Selenium (2-aminoethanesulfonic acid, SE), an essential trace element with antioxidant properties, is involved in successful male reproduction due to its role in testosterone synthesis and development of spermatozoa (Khurana *et al*., 2019). SE plays a key role in spermatogenesis and sperm motility (Das & Ghosh, 2010). Sperm SE content has been positively correlated with the volume of mitochondria in humans and several animal species, such as boars, horses, bulls, and rams (Saaranen *et al*., 1989). SE exists in the outer membrane of the sperm mitochondria as selenoproteins (Calvin *et al*., 1981). Thus, SE deficiency impairs sperm motility and morphology and leads to infertility (Ursini *et al*., 1985). SE improves sperm numbers and motility by selenoproteins (Kaur & Bansal, 2015).

This study looked into the effects of SE on BPA-induced mitochondrial toxicity and impaired mouse sperm motility.

## MATERIALS AND METHODS

### Experimental design (Figure 1)

**Figure 1:**
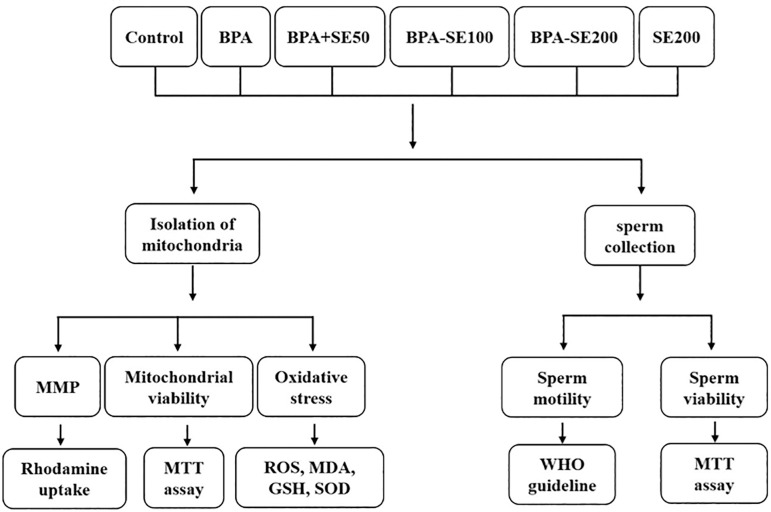
Schematic illustration detailing the experimental design of the study.

Samples of sperm and isolated testicular mitochondria were collected from 48 NMRI mice aged 8-10 weeks. The Ethics Committee on Animal Research approved this study (certificate: ABHC.REC.1397-079).

Spermatozoa were isolated from the epididymis of the mice and divided into six groups, as follows (5×10^6^ sperm/ml were used in each group):

Group I. (Control): treated with medium only

Group II. (BPA): treated with 0.8 mM BPA for two hours

Groups III-V: exposed to 50, 100, and 200 µM SE, respectively, for two hours before exposure to BPA.

Group VI (SE): exposed to 200 µM SE for four hours

The untreated spermatozoa died after four hours. Thus, a total incubation time of four hours was set for exposure of sperms to SE or BPA. BPA (Sigma) or SE (Sigma) was dissolved in dimethyl sulfoxide (DMSO, 0.1%) and diluted in Ham’s F-10 media (Invitrogen). BPA concentrations were based on the results of the MTT test (Table 1). Isolated mitochondria and sperm were exposed to DMSO (1%) for four hours to assess the safety of DMSO by MTT test. DMSO had no toxic effect on isolated mitochondria or mouse sperm (Table 2).

**Table 1:** The effect of different concentrations of BPA on spermatozoa viability

Concentrations	1 hour	2 hours
0 (control)	100.00±0.00	100.00±0.00
0.1 µM	98.4±2.15	95.7±3.23
0.2 µM	92.1±5.41	86.3±4.45
0.4 µM	76.5±5.36	65.9±4.58
0.8 µM	63.9±4.12*	49.8±3.81**
1 µM	56.7±3.72*	35.1±3.09**

Values are expressed as mean ± SD (n=6). *p<0.05, **p<0.01; * comparison against controls.

**Table 2:** Effects of DMSO on testicular mitochondria and spermatozoa

Parameters	Control	DMSO
Viability of sperms (%)	100±0.00	100.01±1.4
Viability of mitochondria (%)	100±0.00	99.5±1.2
MMP (% of control)	100±0.00	100.03±1.1
ROS formation (% of control)	100±0.00	98.9±2.3
MDA of mitochondria (nM/ mg protein)	17.6±5.6	17.4±3.6
GSH of mitochondria (pM/ mg protein)	48.9±2.7	49.1±3.9
SOD of mitochondria (U/ mg protein)	8.8±2.2	8.92±2.4
Total sperm motility (%)	70.2±5.8	70.9±6.5

Values are expressed as mean ± SD (n=6).

### Mitochondria isolation

The testicular tissue of euthanatized mice was dissected and minced in medium containing fat free bovine serum albumin (0.1%), sucrose (250 mM), EGTA (0.2 mM), EDTA (0.1 mM), and HEPES-KOH (5 mM). The minced testicles were homogenized and centrifuged at 3,000·g (10 minutes at 4ºC). The supernatant was centrifuged for 7 minutes at 10,000·g. The prepared mitochondrial fractions were centrifuged for 6 minutes at 10,000·g (twice). The amount of protein was determined using Bradford reagent (Invitrogen). Isolated mitochondria (0.5 mg protein/ mL) was treated with BPA or SE.

### MTT assay

Spermatozoa or mitochondrial fractions were placed in 96-well plates and exposed to SE or BPA. After treatment, 5 mg/ mL MTT (Sigma, USA) was added to the wells and kept for one hour at 37ºC. Then the medium was removed, and DMSO (100 µL) added to each well. A micro-plate reader was used to read absorbance at 570 nm.

### Determining MDA content, ROS formation, and antioxidant levels

The treated mitochondria was poured into the micro-tubes and 10 µM DCFH-DA (Sigma) plus Hank’s buffered salt solution (100 µL) was added and kept at 37ºC for 40 minutes. A spectrofluorometer (LS50B, USA, Em: 570 nm; Ex: 490 nm) was used to examine ROS levels. Protein content of the treated mitochondrial fraction was measured using Bradford reagent (Invitrogen). *Malondialdehyde* (MDA), *superoxide dismutase* (SOD), and GSH (glutathione) activities were evaluated based on the kit’s guidelines (ZellBio Company).

### Mitochondrial membrane potential (MMP) evaluation

The treated mitochondria (0.4 mg protein/mL) was incubated in 10 µM Rhodamine 123 for 15 minutes. A spectrophotometer (LS50B, USA; emission: 535 nm; excitation: 490 nm) was used to measure fluorescence.

### Sperm motility

Sperm motility was evaluated according to WHO guidelines. Sperm suspensions (10 µL) were poured in the semen analysis chamber. Six fields were examined to rate the motility of at least 200 spermatozoa for each specimen. Percent sperm movement was evaluated to estimate the proportions of fast progressive (A), slow progressive (B), no progressive (C), and immotile sperm (D).

### Statistical Analysis

*One-way analysis of variance was performed* in *SPSS* (version 22.0) followed by post-hoc pairwise comparison; *p*-values˂0.05 were deemed significant.

## RESULTS

### MTT assay

**Figure 2:**
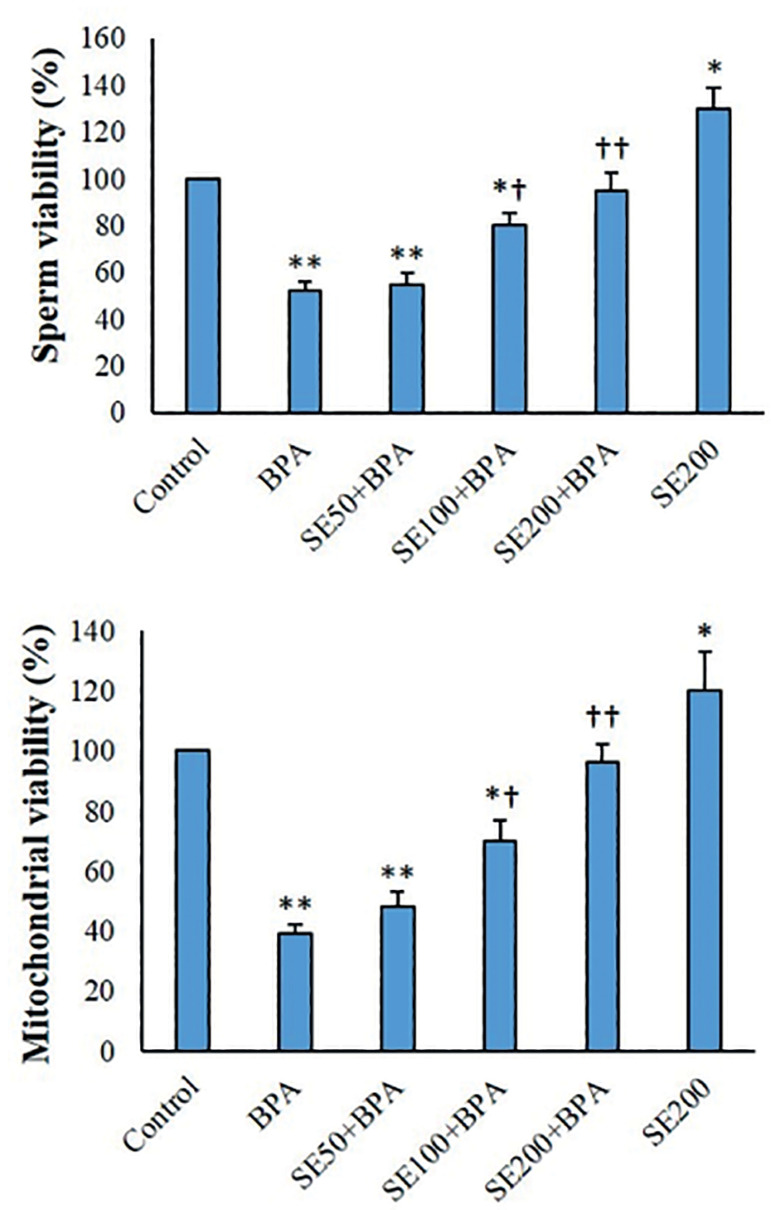
Viability of mitochondrial fractions and spermatozoa (mean ± SD; n=6). ^*^p<0.05, ^**^p<0.01, ^†^p<0.05, ^††^p<0.01; ^*^and ^†^indicate comparisons against untreated control and BPA-treated groups, respectively.

Following exposure to BPA, percent viability decreased considerably in spermatozoa and testicular mitochondria fractions (*p*<0.01). Survival rates grew considerably in the SE-treated mitochondria and spermatozoa (*p*<0.05). SE concentration-dependently elevated survival of the BPA-treated spermatozoa and mitochondrial fractions (Figure 2).

### MDA content, ROS formation, and antioxidant levels

**Figure 3:**
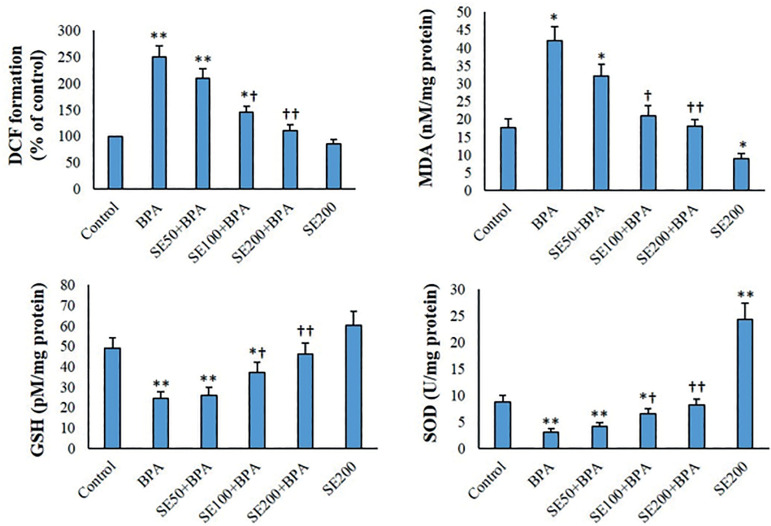
ROS, MDA, GSH, and SOD levels in the mitochondrial fractions (mean ± SD, n=6). *and † indicate comparisons with untreated control and BPA-treated groups, respectively.

Following exposure to BPA, ROS production and MDA levels increased considerably in mitochondrial fractions (*p*<0.01). MDA level and ROS production of the mitochondria decreased in the group treated with SE compared with controls. SE concentration-dependently diminished ROS formation in the BPA-exposed mitochondrial fractions (Figure 3). GSH and SOD activity decreased after exposure to BPA (*p*<0.01). In the SE-treated mitochondria, SOD and GSH activity was higher than the in the untreated (control) group. SE concentration-dependently reversed the BPA-decreased antioxidant level of mitochondrial fractions (Figure 3).

### MMP Assay

**Figure 4:**
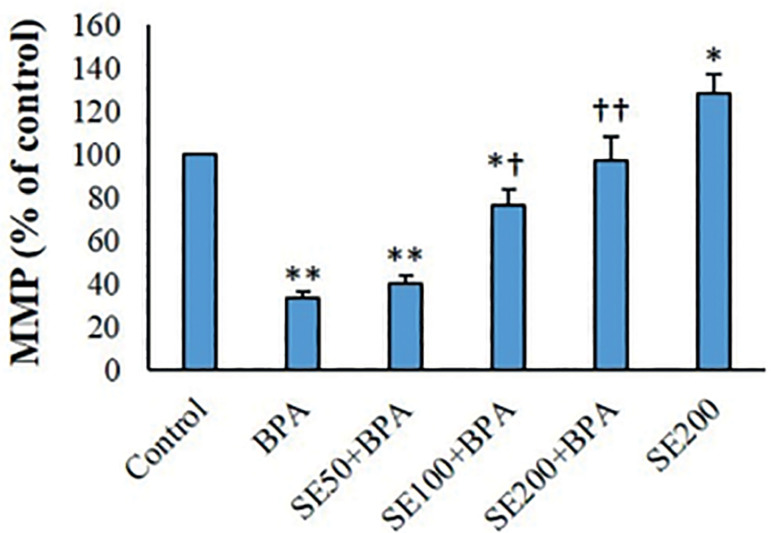
MMP measurement in different groups (mean ^±^ SD, n=6). ^*^ and ^†^ indicate comparisons against untreated control and BPA-treated groups, respectively.

SE significantly increased the MMP of the isolated mitochondria (*p*<0.05). In the BPA-exposed mitochondria, MMP decreased considerably compared with controls (*p*<0.01). SE concentration-dependently elevated the MMP of BPA-exposed mitochondrial fractions (Figure 4).

### Sperm motility

**Figure 5:**
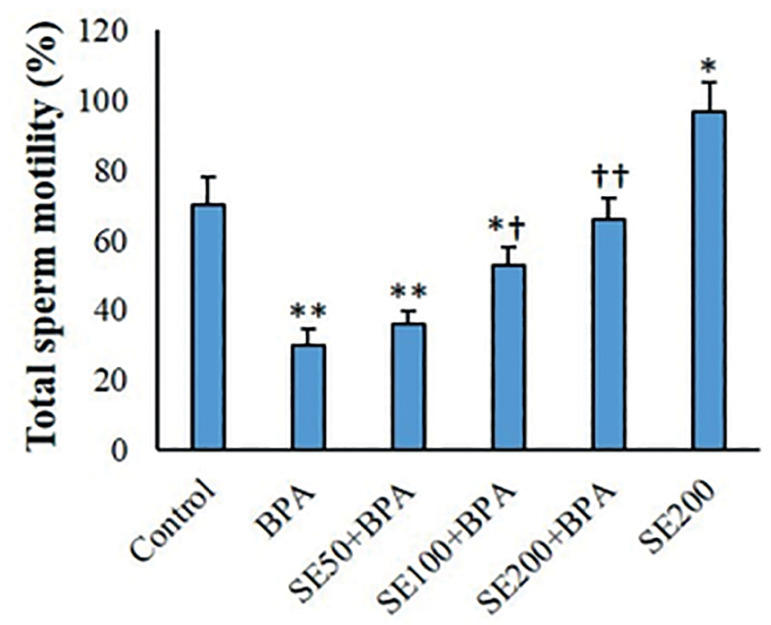
Total mouse sperm motility in the various groups (mean ± SD, n=6). ^*^ and ^†^ indicate comparisons against control and BPA-treated groups, respectively.

SE considerably increased total sperm motility in comparison with controls. Following exposure to BPA, the proportion of fast progressive spermatozoa (*p*<0.05) and total sperm motility (*p*<0.01) diminished considerably, while immotile sperm percentages significantly increased (*p*<0.01). SE concentration-dependently diminished the proportions of immotile and fast progressive sperm, and increased total sperm motility (Table 3 and Figure 5).

**Table 3:** Velocity distribution of sperm cells in various groups

Groups	Fast progressive	Slow progressive	No progressive	Immotile
Control	39.9±4.2	30.3±3.9	16.8±2.5	12.8±2.7
BPA	18.8±2.27**	19.9±2.14*	28.5±3.25*	32.2±2.7**
SE50 + BPA	21.5±3.3**	23.4±2.2	28.1±2.7*	26.6±3.2**
SE100 + BPA	27.8±3.9*^†^	25.1±3.1	24.9±3.4	15.7±2.9^††^
SE200 + BPA	38.7±4.8^†^	22.9±3.8	23.8±3.2	13.9±1.7^††^
SE200	50.3±5.3^††^	30.2±3.6^†^	14.3±2.7	6.2±1.2*^†††^

The mean ± standard deviations are shown (n=6). *p<0.05, **p<0.01, ^†^*p*<0.05, ^††^*p*<0.01, ^†††^*p*<0.001; *and ^†^show comparisons against control and BPA-treated groups, respectively.

## DISCUSSION

The present study demonstrated that SE concentration-dependently reversed the motility and survival of BPA-treated spermatozoa. Consistent with our results, BPA diminished sperm quality in humans and some animal species (Kotwicka *et al*., 2016; Knez *et al*., 2014; Rahman *et al*., 2017).

Rezaeian *et al*. (2016) showed that SE increases the quality of sperm submitted to freezing and thawing procedures. Beneficial effects of SE on Monosodium glutamate-induced spermatogenesis damages have also been reported (Hamza & Diab, 2020).

We observed that SE concentration-dependently enhanced viability, total motility, and fast progressive movement of BPA-exposed mouse sperm cells. In line with our findings, SE provided protection against reproductive system damages induced by zearalenone in male mice (Long *et al*., 2016). SE dramatically altered the changes induced by ethanol on sperm survival and motility to near-normal levels (Swathy *et al*., 2006). SE reportedly reversed the decreased number of spermatogonia and sperm cells induced by radiation (Bagheri *et al*., 2020). Beneficial impacts of SE on the quality of boar sperm have also been documented (Marin-Guzman *et al*., 1997; Bano *et al*., 2018).

As revealed in the results, SE effectively improved the motility of BPA-exposed spermatozoa. Improved motility of spermatozoa might be due to SE effects on mitochondrial mass or function (Shi *et al*., 2010).

This study did not intend to explain the effects SE produced on sperm survival. There is a possibility that SE improves sperm viability by suppressing cell death signaling. Ma *et al*. (2017) reported that SE prevents glutamate-induced cell death in HT22 hippocampal cells. Abu-El-Zahab *et al*. (2019) showed that SE inhibits apoptosis induced by cadmium in mouse hepatic tissue. SE prevented apoptosis induced by lead in chicken nervous tissues (Zhu *et al*., 2017). SE has an important role in the maintenance of follicles and suppression of apoptosis in ovaries (Yang *et al*., 2019). SE inhibits mitochondrial dysfunction and apoptosis in cadmium-induced ROS production within LLC-PK(1) cells (Zhou *et al*., 2009). Kahya *et al*. (2014) showed that mobile phone radiation (900 MHz) induced apoptosis in breast cancer cells through increased oxidative stress. The authors reported that SE attenuated increased oxidative stress and apoptosis.

As mentioned in our results, BPA elevated MDA and ROS levels in testicular mitochondria. In line with our results, BPA increased ROS formation and MDA levels in sperm in previous studies (Rahman *et al*., 2019; Kaur *et al*., 2018). According to our results, SE reversed MMP, ROS production, MDA levels, and antioxidant biomarkers in the BPA-treated mitochondria. Therefore, SE may protect testicular mitochondria by decreasing oxidative stress. It has been shown that SE has a protective effect against mitochondrial oxidative disorders in different pathological cases. These results are in direct correlation with available evidence that indicates the beneficial impacts of SE on mitochondrial activity (Yeo & Kang, 2007; Yoon *et al*., 2002). Neuroprotection provided by SE through reducing ROS production, preventing DNA oxidation, preserving MMP, and mitochondrial function have been reported by Mehta *et al*. (2012).

Ghafarizadeh *et al*. (2018) showed that MMP, sperm survival, total motility, and fast progressive sperm movement of infertile patients were considerably enhanced in SE-exposed samples after four hours of incubation. They concluded that SE protected spermatozoa from mitochondrial damage due to its antioxidant properties.

As shown in our results, BPA decreased the MMP of isolated mitochondria and SE concentration-dependently reversed the effects of BPA on MMP. Barbonetti *et al*. (2016) indicated that BPA diminished MMP and increased human spermatozoa loss. MMP correlates positively with progressive sperm motility and total sperm numbers (Zhang *et al*., 2016). The decrease in MMP caused by BPA was accompanied by increased oxidative stress in testicular isolated mitochondria, reduced sperm motility, and attenuated sperm viability. BPA can cause mitochondrial oxidative damage of testicles via increasing lipid peroxidation (del Hoyo *et al*., 2010), and lead to disruption of spermatozoa function (Catalá, 2009).

SE reduces the glutamate-caused mitochondrial damage and ROS generation in HT22 neuronal cells (Kumari *et al*., 2012). SE has a crucial role in the enzymatic process for the elimination of ROS and helps to preserve membrane integrity. Treulen *et al*. (2016) showed that mitochondrial outer membrane permeabilization induction enhances intracellular ROS and reduces the mean velocity of human sperm cells.

## CONCLUSIONS

Overall, SE concentration-dependently improved MMP and reduced mitochondrial oxidative stress. SE also effectively improved the survival and motility of mouse spermatozoa. It is suggested that SE might ameliorate BPA-caused mitochondrial damage and mouse sperm quality impairment by preventing oxidative stress.

## References

[r1] Abu-El-Zahab HSH, Hamza RZ, Montaser MM, El-Mahdi MM, Al-Harthi WA (2019). Antioxidant, antiapoptotic, antigenotoxic, and hepatic ameliorative effects of L-carnitine and selenium on cadmium-induced hepatotoxicity and alterations in liver cell structure in male mice. Ecotoxicol Environ Saf.

[r2] Anjum S, Rahman S, Kaur M, Ahmad F, Rashid H, Ansari RA, Raisuddin S (2011). Melatonin ameliorates bisphenol A-induced biochemical toxicity in testicular mitochondria of mouse. Food Chem Toxicol..

[r3] Bagheri H, Salajegheh A, Javadi A, Amini P, Shekarchi B, Shabeeb D, Eleojo Musa A, Najafi M (2020). Radioprotective Effects of Zinc and Selenium on Mice Spermatogenesis. J Biomed Phys Eng.

[r4] Bano I, Malhi M, Soomro S, Kandhro S, Awais M, Baloch S, Perveen, Sajjad H (2018). Effect of Dietary Selenium Supplementation on Morphology and Antioxidant Status in Testes of Goat. J Basic Appl Sci.

[r5] Barbonetti A, Castellini C, Di Giammarco N, Santilli G, Francavilla S, Francavilla F (2016). In vitro exposure of human spermatozoa to bisphenol A induces pro-oxidative/apoptotic mitochondrial dysfunction. Reprod Toxicol.

[r6] Catalá A (2009). Lipid peroxidation of membrane phospholipids generates hydroxy-alkenals and oxidized phospholipids active in physiological and/or pathological conditions. Chem Phys Lipids..

[r7] Calvin HI, Cooper GW, Wallace E (1981). Evidence that selenium in rat sperm is associated with a cysteine-rich structural protein of the mitochondrial capsules. Gamete Res.

[r8] Chitra KC, Latchoumycandane C, Mathur PP (2003). Induction of oxidative stress by bisphenol A in the epididymal sperm of rats. Toxicology.

[r9] Das RS, Ghosh SK (2010). Long term effects of monosodium glutamate on spermatogenesis following neonatal exposure in albino mice--a histological study. Nepal Med Coll J..

[r10] del Hoyo P, García-Redondo A, de Bustos F, Molina JA, Sayed Y, Alonso-Navarro H, Caballero L, Arenas J, Agúndez JA, Jiménez-Jiménez FJ (2010). Oxidative stress in skin fibroblasts cultures from patients with Parkinson’s disease. BMC Neurol.

[r11] Engel LS, Buckley JP, Yang G, Liao LM, Satagopan J, Calafat AM, Matthews CE, Cai Q, Ji BT, Cai H, Engel SM, Wolff MS, Rothman N, Zheng W, Xiang YB, Shu XO, Gao YT, Chow WH (2014). Predictors and variability of repeat measurements of urinary phenols and parabens in a cohort of Shanghai women and men. Environ Health Perspect.

[r12] Ghafarizadeh AA, Vaezi G, Shariatzadeh MA, Malekirad AA (2018). Effect of in vitro selenium supplementation on sperm quality in asthenoteratozoospermic men. Andrologia.

[r13] Hamza RZ, Diab AEA (2020). Testicular protective and antioxidant effects of selenium nanoparticles on Monosodium glutamate-induced testicular structure alterations in male mice. Toxicol Rep.

[r14] Ikezuki Y, Tsutsumi O, Takai Y, Kamei Y, Taketani Y (2002). Determination of bisphenol A concentrations in human biological fluids reveals significant early prenatal exposure. Hum Reprod.

[r15] Kahya MC, Nazıroğlu M, Çiğ B (2014). Selenium reduces mobile phone (900 MHz)-induced oxidative stress, mitochondrial function, and apoptosis in breast cancer cells. Biol Trace Elem Res.

[r16] Kamali Sangani A, Masoudi AA, Vaez Torshizi R (2017). Association of mitochondrial function and sperm progressivity in slow- and fast-growing roosters. Poult Sci..

[r17] Kaur S, Bansal MP (2015). Protective role of dietary-supplemented selenium and vitamin E in heat-induced apoptosis and oxidative stress in mice testes. Andrologia.

[r18] Kaur S, Saluja M, Bansal MP (2018). Bisphenol A induced oxidative stress and apoptosis in mice testes: Modulation by selenium. Andrologia.

[r19] Khurana A, Tekula S, Saifi MA, Venkatesh P, Godugu C (2019). Therapeutic applications of selenium nanoparticles. Biomed Pharmacother.

[r20] Knez J, Kranvogl R, Breznik BP, Vončina E, Vlaisavljević V (2014). Are urinary bisphenol A levels in men related to semen quality and embryo development after medically assisted reproduction?. Fertil Steril.

[r21] Konieczna A, Rutkowska A, Rachoń D (2015). Health risk of exposure to Bisphenol A (BPA). Rocz Panstw Zakl Hig.

[r22] Kotwicka M, Skibinska I, Piworun N, Jendraszak M, Chmielewska M, Jedrzejczak P (2016). Bisphenol A modifies human spermatozoa motility in vitro. J Med Sci..

[r23] Kumari S, Mehta SL, Li PA (2012). Glutamate induces mitochondrial dynamic imbalance and autophagy activation: preventive effects of selenium. PLoS One.

[r24] Löfroth M, Ghasemimehr M, Falk A, Vult von Steyern P (2019). Bisphenol A in dental materials - existence, leakage and biological effects. Heliyon.

[r25] Long M, Yang S, Wang Y, Li P, Zhang Y, Dong S, Chen X, Guo J, He J, Gao Z, Wang J (2016). The Protective Effect of Selenium on Chronic Zearalenone-Induced Reproductive System Damage in Male Mice. Molecules.

[r26] Ma YM, Ibeanu G, Wang LY, Zhang JZ, Chang Y, Dong JD, Li PA, Jing L (2017). Selenium suppresses glutamate-induced cell death and prevents mitochondrial morphological dynamic alterations in hippocampal HT22 neuronal cells. BMC Neurosci.

[r27] Marin-Guzman J, Mahan DC, Chung YK, Pate JL, Pope WF (1997). Effects of dietary selenium and vitamin E on boar performance and tissue responses, semen quality, and subsequent fertilization rates in mature gilts. J Anim Sci.

[r28] Mehta SL, Kumari S, Mendelev N, Li PA (2012). Selenium preserves mitochondrial function, stimulates mitochondrial biogenesis, and reduces infarct volume after focal cerebral ischemia. BMC Neurosci.

[r29] Mikołajewska K, Stragierowicz J, Gromadzińska J (2015). Bisphenol A - Application, sources of exposure and potential risks in infants, children and pregnant women. Int J Occup Med Environ Health.

[r30] Rahman MS, Kwon WS, Karmakar PC, Yoon SJ, Ryu BY, Pang MG (2017). Gestational Exposure to Bisphenol A Affects the Function and Proteome Profile of F1 Spermatozoa in Adult Mice. Environ Health Perspect.

[r31] Rahman MS, Kang KH, Arifuzzaman S, Pang WK, Ryu DY, Song WH, Park YJ, Pang MG (2019). Effect of antioxidants on BPA-induced stress on sperm function in a mouse model. Sci Rep.

[r32] Rezaeian Z, Yazdekhasti H, Nasri S, Rajabi Z, Fallahi P, Amidi F (2016). Effect of selenium on human sperm parameters after freezing and thawing procedures. Asian Pac J Reprod.

[r33] Saaranen M, Suistomaa U, Vanha-Perttula T (1989). Semen selenium content and sperm mitochondrial volume in human and some animal species. Hum Reprod.

[r34] Shi L, Yue W, Zhang C, Ren Y, Zhu X, Wang Q, Shi L, Lei F (2010). Effects of maternal and dietary selenium (Se-enriched yeast) on oxidative status in testis and apoptosis of germ cells during spermatogenesis of their offspring in goats. Anim Reprod Sci..

[r35] Swathy SS, Panicker S, Indira M (2006). Effect of exogenous selenium on the testicular toxicity induced by ethanol in rats. Indian J Physiol Pharmacol.

[r36] Treulen F, Uribe P, Boguen R, Villegas JV (2016). Mitochondrial outer membrane permeabilization increases reactive oxygen species production and decreases mean sperm velocity but is not associated with DNA fragmentation in human sperm. Mol. Hum Reprod.

[r37] Tyl RW, Myers CB, Marr MC, Sloan CS, Castillo NP, Veselica MM, Seely JC, Dimond SS, Van Miller JP, Shiotsuka RN, Beyer D, Hentges SG, Waechter Jr JM (2008). Two-generation reproductive toxicity study of dietary bisphenol A in CD-1 (Swiss) mice. Toxicol Sci..

[r38] Ullah A, Pirzada M, Jahan S, Ullah H, Shaheen G, Rehman H, Siddiqui MF, Butt MA (2018). Bisphenol A and its analogs bisphenol B, bisphenol F, and bisphenol S: Comparative in vitro and in vivo studies on the sperms and testicular tissues of rats. Chemosphere.

[r39] Ursini F, Maiorino M, Gregolin C (1985). The selenoenzyme phospholipid hydroperoxide glutathione peroxidase. Biochim Biophys Acta.

[r40] Yang H, Qazi IH, Pan B, Angel C, Guo S, Yang J, Zhang Y, Ming Z, Zeng C, Meng Q, Han H, Zhou G (2019). Dietary Selenium Supplementation Ameliorates Female Reproductive Efficiency in Aging Mice. Antioxidants (Basel).

[r41] Yeo JE, Kang SK (2007). Selenium effectively inhibits ROS-mediated apoptotic neural precursor cell death in vitro and in vivo in traumatic brain injury. Biochim Biophys Acta.

[r42] Yoon SO, Kim MM, Park SJ, Kim D, Chung J, Chung AS (2002). Selenite suppresses hydrogen peroxide-induced cell apoptosis through inhibition of ASK1/JNK and activation of PI3-K/Akt pathways. FASEB J.

[r43] Zhang G, Wang Z, Ling X, Zou P, Yang H, Chen Q, Zhou N, Sun L, Gao J, Zhou Z, Cao J, Ao L (2016). Mitochondrial Biomarkers Reflect Semen Quality: Results from the MARCHS Study in Chongqing, China. PLoS One.

[r44] Zhou YJ, Zhang SP, Liu CW, Cai YQ (2009). The protection of selenium on ROS mediated-apoptosis by mitochondria dysfunction in cadmium-induced LLC-PK(1) cells. Toxicol In Vitro.

[r45] Zhu Y, Jiao X, An Y, Li S, Teng X (2017). Selenium against lead-induced apoptosis in chicken nervous tissues via mitochondrial pathway. Oncotarget.

